# The Physiological Antagonism of Remedies

**Published:** 1881-06

**Authors:** 


### THE PHYSIOLOGICAL ANTAGONISM OF REMEDIES.

The fact that the physiological antagonism of remedies has recently been the subject of increased scientific attention, has a practical significance, in so far as the questions therein involved promise, in their solution, a varied and substantial benefit. The subject has been in a more or less crude state for a period up to the era inaugurated principally by Fothergill and Bartholow, who gave it much impetus by their labors and researches.

The subject matter of the observations on this question are discussed from a clinical standpoint, while, probably of necessity, the physiological mechanism of the various phenomena observed receive but meagre attention. Should anyone attempt to explain on purely physiological grounds the antagonistic influences existing between morphine and atropia, or any other of the antagonistic alkaloids, the practical understanding would, very apt, be confounded or lost in the labyrinth of theory. But many facts have been clinically demonstrated, and these serve as landmarks in the prosecution of the enquiry.

It would be well for every one to report any personal experience relevant to the subject, since in this way details may accumulate that will indicate some substantial fact.

The writer had once occasion to observe an apparent antagonism between chloroform and strychnine. The exhibition of the latter, in connection with whisky hypodermically, in a case of poisoning by the former, resulted in recovery where dissolution appeared to be inevitable. Whether or not this supposed antagonism would be confirmed by further observation is a question that we would gladly see demonstrated.

